# Dr. Pring's Exposition of the Principles of Pathology and Therapeutics

**Published:** 1823-09-01

**Authors:** 


### Medico-Chirurgical Review,

AND

### JOURNAL OF MEDICAL SCIENCE.

(Analytical Series.)

"Nec Araneаrum textus ideò melior, quia ex se fila fingunt; nec noster vilior, quia ex alienis libamus, ut apes."

VOL. IV.] SEPTEMBER 1, 1823. [No. 14.

### I.

*An Exposition of the Principles of Pathology, and of the Treatment of Diseases.* By DANIEL PRING, M.D. Member of the Royal College of Surgeons, London. Octavo, pp. 512. London, April 1823.

IF the writings of Mr. now Dr. Pring be not familiarly known to the profession, it certainly is not for want of their being analyzed, criticised, and advertised in journals, reviews, and newspapers. Our former cotemporary, Dr. Hutchinson, proclaimed the "Laws of Organic Life" to be the sublimest production of the age in which we live—and the author not to be inferior to a Locke, or even a Newton. To the disgrace of this age, the work lies mouldering on the shelves of the booksellers. In the third number of this Journal, for December 1820, we took the liberty of exposing the atheistical principles so freely broached, or rather openly avowed, in Dr. Pring's work, and of shewing how dangerous, how unnecessary, and how uncalled-for was this incorporation of atheism and materialism with the principles or the practice of medicine. We are not without hopes that we have been instrumental in checking this propensity among our modern medical writers—and that so far we have been useful in our calling. But we acknowledged the author's talents, while we condemned his metaphysics—and we cannot think we deserved, in so doing, the epithet "pugnacious," which has been applied to us by a cotemporary.*

* An admiring reviewer of Dr. Pring, in the Literary Register for May 10th, 1823, substantially admits that we advocated a just cause,

We are not vain enough to suppose that our animadversions made any impression on Dr. Pring himself; but whether or not, he has laid aside his metaphysical speculations and atheistical principles in the work before us, and kept to his text. On this account we enter on our task of analysis or criticism, without the slightest prejudice against the author; but, on the contrary, with a bias in his favour. We will analyse fairly, and criticise freely, if we see occasion; but we will "nothing extenuate, nor set down aught in malice."

We confess, indeed, that we were somewhat staggered at Dr. Pring's declaration in the preface that so important a work as the principles of pathology and therapeutics, was begun and finished during a summer tour of a *few months*, and that without other library than "*an odd volume or two of Shakespeare and of Don Quixote.*" Whether in this Cervantic expedition the example of La Mancha's knight may have led our author to mistake windmills for giants, or exercise his toledo or pewter basons instead of Membrino helmets—or whether, on the other hand, the genius of Shakespeare may have led him sometimes to "turn physic to a farce," will be seen as we proceed. For the task of this last conversion it must be allowed that our author is peculiarly well qualified, being a sceptic in principle, and primed to the muzzle with logical distinctions and metaphysical subtleties. With far less of these acquirements, indeed, than Dr. Pring possesses, any man of talent, and so inclined, might contrive to make the whole science of medicine appear a chaos of absurdity, contradiction—or, at least, of doubt. Many of our best principles (which, though erroneous, may be necessary, and even useful, in practice) might thus be turned into ridicule, and the youthful mind left to wander without guide, and indeed without hope, "through all the

---

and not without effect. "We despaired," says the Reviewer, "of the frail support it might derive from our criticism, and with a truly honorable reserve, we distrusted our capability of doing justice to its (the work now under review) philosophic and speculative spirit—to its depth and occasional originality of thought—to its fulness of detail—its felicity of expression—and the manly idiomatic strength of its style. We forgot, for a while, that the powers and attainments of a mind, gifted like his, had been *basely sacrificed to the foul and pestilential doctrines on which we forbear to set a brand*; and we hoped that the *most pugnacious* of all critics, the mighty men in panoply of blue, would fling aside the hostile weapon, and give unto Cæsar that which they had so earnestly and severely exacted for God."

We *did* give unto Cæsar *that which was Cæsar's*; but we have yet to learn why we should also have given him *that which was God's*.

mazes of physical and metaphysical confusion." Such would assuredly be the way to retard, not advance the science; and although many portions of Dr. Pring's writings have this tendency, we freely absolve him of all intention to produce such effects. His chief object is unquestionably to demolish error; but in medicine there is generally some truth mixed up with error, and the broom of correction, if too freely applied, may sweep away the good and bad indiscriminately.

Of a truth, it is much more easy to pull down an old house than build up a new one; and therefore Dr. Pring has chosen the more facile task of levelling systems already tottering or in ruins, rather than construct another to sink in turn by its own weakness, or the novelty of a rival. And yet it seems high time for some genius to start and erect the edifice of Hypothesis, if the following picture be correct, which we are not disposed to deny.

> "It cannot be said, at this time, that any one system of medicine prevails exclusively; or scarcely, that of the various ones extant, one is more extensively adopted than another. Indeed, so unsettled is the state of pathology, that those who read, are sceptics in all its doctrines; and those who do not read, are left to the guidance of a sort of intuition, which is not always productive of happy results; but very frequently suggests, through the course of a long life, only a reiteration of the same errors." *Pref.* p. 1.

This being the case, it appeared desirable to our author to take a compendious review of certain questionable doctrines, in order to expose a few errors and establish a few truths—so as to lay some kind of stable foundation for a future system of pathology amid this desolate waste of wrecks and ruins.

The humoral pathology claims our author's first notice, but not at a higher date of its existence than the days of Boerhaave, by whom it "may be said to have been perfected." He does not deem it necessary (and we think him right) to criticise the four humours of Hippocrates, the doctrines of the magi, the heat and moisture of Galen, or the four winds of Paracelsus.

It is easy also to conceive that our ingenious author has made sad havoc among those portions of the Boerhaavian doctrine which attempted the explanation of animal phenomena by conflicts and harmonies, spherical and angular particles, calibres, remoræ, errores loci, salts, oils, and acrimonies. And yet he says the evidences in favour of this doctrine "are quite as conclusive" as those which support some of the doctrines of the present day. In fact, he acknowledges, and not without reason, that many parts of the humoral pathology are not without truth for their basis.

It is well known, to our cost indeed, that certain animal fluids, the products of disease, for instance the morbid poisons as they are called, are capable of producing similar diseases in other subjects. The *way* in which they produced their effects, as explained by the humoralists, is indeed sufficiently unsatisfactory if not ridiculous, and our author has set about an explanation upon other principles than those of mechanics—with what success the reader will soon see by the following extract:—

"It appears then, in the case of the peccant humours, that their phenomena are not produced by any mechanical agency. It is more agreeable with the results of analytical inquiry to conclude, that the animal poisons contain latent properties of a vital kind, which are related with those of the same kind in living bodies; that the phenomena of disease or death, which ensue from the operation of the animal poisons on living bodies, are according to the nature of the properties which are engaged in this relation. It seems proper, in all questions which respect the nature of the properties to which animal processes are to be assigned, to consider those agents as belonging to the department of life, whose operation cannot be explained by any perceptible analogy to the agency of chemistry or of mechanism.

"It appears then, that certain animal fluids are endowed with the qualities of poisons, by latent vital properties which are allied with the fluid material. This affinity between these subtle properties and the grosser fluid, prevents the dissipation of the former, and preserves also the characteristics of the latter; it serves at once to limit and to extend the relations of the poisonous qualities: by it these qualities have a fixed place; and by the medium with which they inhere, they are capable of mingling with the fluids of animal bodies, and of being, in this way, conveyed into the circulation. It appears probable, that by the existence or absence of this affinity between the active properties of the animal poisons, and a fluid medium, the distinction may be furnished between the contagious and the infectious diseases. According to the entire absence, or the degree of this affinity, we should find some diseases which are wholly infectious, others which are both infectious and contagious, and others which are only contagious." 11.

Thus, then, if we understand the Doctor's theory, these morbid poisons do not enter the system as foreigners or enemies, but as relations allied together by the bonds of vitality! Really we see no more lucid explanation of their modus agendi on this principle, than on the old and exploded one of "*peccant matter.*"

In respect to *errores loci*, our author thinks it quite impossible that such a thing, that is, an obstructed state of the vessels, should occur in any other way than by means of the secretions from the vessels themselves—or by blood being forced into the capillaries. In either case he does not admit that the fluids, whether blood or secretions, are ever stagnant in the containing vessels. Thus in the most injected state of the conjunctival vessels in ophthalmia, the blood, he avers, is not at rest. Our author takes up some time to prove that biliary obstructions cannot arise, in any case, from the bile itself, but from some wrong action in the secretory structure of the liver—and so on, of other secreted fluids, about which our author is very knowing and very unintelligible.

Lentor and viscidity of the blood our author does not deny, since our senses shew us great differences in these respects.

"That which has occurred to me in this respect, must also have been generally remarked by others. It is no uncommon circumstance to find blood taken from a vein of a very dark colour, and flowing almost like thin treacle, or pitch. Such blood may be remarked, upon separating, to contain but a very small portion of serum, and a crassamentum in general of weak cohesion. On the other hand, it is common to find venous blood, which consists of a small proportion of crassamentum, which, if the blood is uninflamed, is also of weak cohesion, and a very large proportion of serum." 19.

Whether a treacly blood may require a greater exertion of the heart to give it velocity than a watery blood requires, we do not pretend to know, though our author pronounces peremptorily in the affirmative, without an atom of proof that we can see. But be this as it may, we certainly did not expect to find a man, who discarded hydraulics from his system, making use of so mechanical an explanation as that of *thick* blood causing vertigo by its *weight* in the vessels of the brain.

"The person in whom I remember this state of the blood to have existed in the greatest degree, was dyspeptic, subject to vertigo, and temporary confusion, if not alienation, of mind.

"The symptoms just mentioned could not arise from any relation between this state of the blood and the action of the heart: *it is not, however, impossible that the brain, the function of which is so highly susceptible of the influence of pressure, may suffer by this mode, from the preternatural weight of the blood in its vessels.*" 21.

If this be not mechanical philosophy applied to the living system we know not what is. It is somewhat curious, that *too thin blood* affects the head nearly in the same way as the *heavy blood*, which is rather unfavourable, we imagine, to the Doctor's theory.

"It is commonly, and in my own experience it has been I believe invariably, the case, that those who have sustained great losses of blood, suffer more or less from what is called determination to the head. The symptoms most commonly are intense pain, and throbbing in the forehead or back part of the head, with a pulse seldom under ninety." 23.

We agree with Dr. Pring that the dropsical effusions which sometimes succeed profuse losses of blood, whether by accident or design, are not to be accounted for by the hydraulic explanation of the watery parts of the blood running off freely through the secerning extremities of the arteries. It is far more rational to suppose that the phenomenon depends on some derangement of function in the secerning powers, or vital properties of the vessels.

DOCTRINE OF SPASM.

Notwithstanding Dr. Pring's disheartening sentiment at the commencement of this chapter, namely, that "in the more theoretical sciences it often happens that the career of improvement is marked by the substitution of a new error in place of an old one," we think the doctrine of spasm and nervous agency in fevers and other diseases was an immense stride towards truth. In short, we consider many of Dr. Pring's strictures on the Cullenian doctrine as very futile, or, at the best, but ingenious sophistry. Take the following passage for an example.

"That the mere suppression of perspiration is a cause sufficient to produce fever, is an opinion which is generally admitted. The correctness of it will however be doubted, when we consider the frequency of the occurrence. It happens to most individuals, perhaps every day during the summer, in a greater or less degree; and is strikingly the case with those whose occupations subject them to sudden and extreme transitions of temperature; from the forge, the glass-furnace, the bakehouse, &c. to an atmosphere perhaps below thirty. But it will be remarked in these instances, the perspiration suffers only a temporary check; it returns with the recurrence of labour, or with the exposure to a higher temperature. In many of those examples of disease also, which are ascribed to suppressed perspiration, the phenomena of the disease continue after the perspiration has been restored, even more copiously than before. This is particularly observable in acute rheumatism, in pneumonia, and in some local inflammations connected with the puerperal state, which are ascribed to cold, as inflammation of the breasts, legs, and even peritonitis: in all which cases, I have seen, no doubt in common with many others, patients appearing as if boiled in sweat, while the disease has run a protracted course, perhaps, though rarely, to a fatal termination." 34.

Thus because glass-blowers and bakers, from long habit, are able to bear great transitions of temperature without fever being the consequence, we are to draw a general conclusion that all other people may experience the same thing with perfect impunity. And again, because we see patients, in a certain stage of febrile diseases, "appearing as if boiled in sweat," we are therefore to infer that the *natural and healthy functions* of the skin are perfectly restored, and consequently that its previously suppressed or deranged functions had nothing to do in the production of the disease. This is admirable reasoning, and very well calculated for a debating society, where the establishing of a truth is quite subordinate to the supporting of an argument.* The following quotation will shew Dr. Pring's talent for splitting hairs, and proving that causes are effects and effects causes.

"But the production of the phenomena which have been ascribed to checked perspiration, is liable to the alternatives of two modes; perhaps a third might be added. If the entrance of fluids into the extensive system of cutaneous vessels is resisted by a preternatural contraction of these vessels, it is obvious that those vessels which are not cutaneous must contain more than their natural proportion of fluids: this state would be one of general internal plethora. But we observe that, as a consequence of suppressed perspiration, one organ always suffers principally; that there is a particular determination to one seat. If a contracted state of the cutaneous vessels could throw an unnatural proportion of fluids into the internal ones, so preternatural dilatation of the vessels of any other seat or system would deprive the cutaneous vessels of their natural proportion. We have then to choose whether disease is maintained by suppressed perspiration, or whether the suppression of perspiration is a consequence of disease.

"If we consider here merely the order of occurrence, it would appear that the disease, say, for example, acute rheumatism—was produced by suppressed perspiration. At the same time, there is no reason why the muscles, rather than any other parts, should suffer from the suppression of perspiration; seeing that the simple circumstance of a diminished circulation in the skin is equally relative to the vascular system of every seat. In order that one seat should suffer a vicarious determination rather than another, it is necessary to suppose a predisposition in such seat which does not exist in others. This predisposition is very nearly allied to disease; and the admission of it goes near to confessing that the circumstance of a suppressed perspiration was preceded by processes of disease in that seat which afterwards exhibits its chief phenomena." 36.

* Whatever importance Cullen himself may have attached to the *mere suppression* of *perspiration*, it is well known that, since the time of Darwin especially, the sympathetic affections of the interior organs, in consequence of the deranged functions of the skin, formed the prominent object of contemplation. But it suits our author's purpose not to notice the Darwinian theory, but to set up the weakest points of the Cullenian, as foils for his own prowess. This is the way with many people, who, like Johnson of old, argue for the purpose of conquering, not convincing.

Now all this puzzling and subtlety only goes, in our apprehension, to shew, what has been long known, that when cold or wet is applied to the skin, and becomes a cause of fever or inflammation, the weakest organ or structure suffers the most. We do not think it worth our while to analyze any farther Dr. Pring's criticisms on the doctrine of spasm. We have not been able to profit by them ourselves, and it is very improbable that we should be able, through their medium, to edify our readers.

Dr. Pring passes from Cullen to Brown, without noticing Darwin, whose theories and practice were superior to all who went before him, and all who have followed him. We suspect that our author found it prudent not to meddle with Darwin, whose perfect acquaintance with every form, feature, and phenomenon of disease at the bed-side, gave him a decided superiority over book-practitioners and theoretical speculators, whose errors and inconsistencies Dr. Pring found it easier to expose. He has accordingly taken up a great many pages in the review of the exploded doctrines of John Brown, through which review we certainly shall not attempt to follow our critic. Speaking of the few good points in the Brunonian theory, he observes that there is one practice arising out of it which is worth retaining, namely, the practice of increasing very slowly the means of repletion after considerable privations or depletions.

"In the beginning of convalescence from fever, I have known a single meal of a nutritious or stimulating quality, to which the system had been long unaccustomed under the previous treatment of abstinence and depletion, followed by apoplexy, which was fatal in a few hours. Nothing also is more common than violent determinations of blood to the head by sudden repletion after a copious loss of blood, whether from accidental hemorrhage, or by the frequent use of the lancet. I have known such determinations to be frequently produced by this cause in puerperal states; and the effects, of which this determination was the cause or the accompaniment, have exhibited every degree of cerebral disorder, from mere pain and throbbing in the head to delirium, epilepsy, or apoplexy: and I have known the first stimulating meal taken, contrary to direction, twelve days after a delivery which was attended with profuse flooding, followed immediately by accelerated pulse, pain in the head, eventually coma, and death. In a similar case, a single glass of porter, given injudiciously four days after delivery, has been immediately followed by apoplexy; in which state the woman remained for several hours, and from which she recovered with difficulty. These cases might be greatly multiplied; but this seems superfluous, as the fact does not stand in need of additional examples.

"It is common, in these severe and threatening forms of cerebral disorder, notwithstanding previous loss of blood, to resort to the lancet, and to repeat the bleeding, copiously and frequently, if the symptoms continue. So far as I have had opportunities of observation, this practice carried to any great extent, to the neglect perhaps of other measures, has generally had a fatal termination. I have been in the habit of confiding in purgatives, to the almost total neglect of the lancet; but these purgatives have not been of a milk and water kind. If, after such copious loss of blood, the pulse has suddenly got up from eighty to a hundred and thirty or forty in a minute, with intense pain in the head, perhaps slight incoherence of speech, restlessness, &c. I have given six grains of calomel, and the same quantity of James' Powder, followed by a two-ounce draught of salts and senna, with half a dram of jalap in it. If this has been rejected, it has been repeated in less than an hour, and repeated as often as it was rejected, until it has produced copious evacuations from the bowels. One, and perhaps an important, effect of such purgatives, is to make a great revulsion to the whole intestinal canal; which is commonly followed by almost perfect relief of the head, and perhaps an immediate subsidence of the pulse to a hundred, or a hundred and ten. The tendency to disorder of the head is afterwards easily kept within safe bounds, by small, but repeated doses of Epsom salts; or an exacerbation, if it occurs, may be reduced by a repetition of the former purgatives. If, notwithstanding a full and continued purging, the disorder should persevere in its course, I have reason to think, from the results of cases essentially analogous, that putting the system suddenly under the full influence of mercury is more likely to subvert the disposition to cerebral disorder than the employment of the lancet. But before this measure is resorted to, the fatal termination of the case (unless a vigorous effort is made to avert it) must be so decidedly indicated, that the further prosecution of curative endeavours seems little better than a forlorn hope. In a pretty considerable experience, I have never met with a fatal instance of disorder of the head of this kind when, however threatening and severe the symptoms might be, the plan of treatment by purgatives, nauseating, and perhaps emetic remedies has been wholly or chiefly depended upon. And, so far as my recollection serves me, all the fatal cases which have come to my knowledge have been those in which the treatment of disorder of the head of this kind has been rested wholly or principally upon the use of the lancet." 86.

The baleful influence of the Brunonian theory on practice, leads our author to make some very good remarks on the treatment of fever. It rarely happens, he thinks, that fever is unattended with local inflammation of some important viscus. "In typhus the brain perhaps invariably suffers in a greater or lesser degree, either from determination of blood, from inflammation, or congestion." It is well known that stimuli, especially the alcoholic stimuli, have a tendency to produce or augment these determinations, whether cerebral or visceral; and we coincide entirely in the following sentiment of our author—a sentiment, by the bye, which has been often expressed by ourselves before Dr. P. wrote.

"I apprehend it is generally, though perhaps not invariably true, that if visceral inflammation or congestion in fever can be kept within safe bounds, very little is to be feared from the constitutional state; and that the disease will almost, without an exception, run a safe, though perhaps a protracted course." 89.

He thinks that it is only in the advanced stages of what is denominated typhus gravior in this country, that stimuli can be used with safety or benefit—and, moreover, he rationally conjectures that if cases of ordinary typhus were properly treated in the beginning, we should have very few specimens of the typhus gravior to combat. Our author gives his testimony in support of the opinion, (always maintained by us,) "that typhus has two origins—one from external infection, and the other from a spontaneous generation of the disease in the subject affected by it." Dr. Pring commends moderate bleeding, especially in the beginning of typhus; but his favourite practice is purgation of a very active kind.

"When, in spite of the most efficient treatment by bleeding in the beginning, purgatives, &c. typhus has run into the state of coma, and a fatal termination seemed inevitable, and weaker measures have been of little promise, I have found it a successful effort to put the patient rapidly under the influence of mercury, which I have done, by giving ten grains of calomel every six or eight hours. I have known all the alarming symptoms cease as soon as the saliva began to flow freely from the mouth of the insensible patient upon the pillow; and the convalescence has been regular from that period. A boy, fourteen years of age, whose case was a neglected one, who had been ill of typhus a fortnight without the employment of medicine of any kind, and who had gone progressively from fever, with determination of the head, into a complete state of coma, recovered his sensibility, and understood what was said to him in thirty-six hours, under a salivation, produced in this time, by large doses of calomel and mercurial frictions; he made many efforts to speak, at first unsuccessfully, and it was nearly a week before the power of articulation was restored. This boy recovered so perfectly, as to be no longer under medical care. His appetite afterwards became voracious; and owing, most probably, to an imprudent indulgence of it, he had apoplexy, which occurred soon after a meal, and died in a few hours.

"I have also known coma and insensibility to occur after a three months' course of typhus, beginning with the atonic form, and becoming inflammatory in its progress. The death of this patient (a girl about twenty years of age) was expected every hour. As a last effort I gave her ten grains of calomel every six hours; and although strabismus accompanied the insensibility, in so great a degree that very little more than the conjunctiva of either eye was visible, she began to recover as soon as the saliva ran from her mouth, which occurred on the second day of this treatment; she eventually regained a perfect state of health.

"It appears to me, that the state here described, or one nearly approaching to it, is the proper stage for the employment of mercury, with a view to a complete constitutional affection by this remedy. At the same time, its employment with this view is not limited to this stage: it is a powerful means of arresting tendencies of disease which threaten to be fatal, if not effectually checked; when, from the character, or degree of the symptoms, the more common remedies appear inadequate to this end. Among other modes of the curative operation of mercury, perhaps the determination to the whole glandular system, of which effect salivation may be considered only as a part, or as the indication of a more general affection of this system, comprising the liver, kidneys, &c. together with the skin, is by no means the least important; and an influence by mercury, short of this effect, would, in extreme cases, be most probably useless. This general affection of the glandular system is scarcely less positively indicated by the circumstance of salivation than by the stools, which consist commonly, under the mercurial affection, or soon after it, of depraved hepatic secretions of almost every variety. The surface of what might be called counter-determination, is also greatly extended by the action of mercury upon the intestinal canal." 102.

Of the stimulating treatment of typhus, our author has little experience, and the less the better. Yet he has "seen patients stimulated into fatal apoplexy, or suffered to go quietly into this state." He mentions a remark of Dr. Falconer of Bath, and corroborates it by his own experience, that subsultus tendinum in fever is invariably a fatal symptom. We certainly have seen patients recover after this symptom had appeared, though it undoubtedly is a very unfavourable omen.

The fourth chapter is on the "*pathology of the determination of blood*"—a term in medicine of no modern date, although now more frequently used than formerly, because the doctrine connected with it is more completely organized. Frederick Hoffman, indeed, was reproached for having made local determination of blood the cause of fever, of inflammation, of spasmodic, and of most other diseases. There is, therefore, no novelty in the doctine.* It is evidently, however, to the late Dr. Parry that we are indebted for all the perfection which this doctrine of determination has yet obtained.

* It is astonishing how much of all the new doctrines and ideas are to be found in the works of Hoffman—especially the Abernethian doctrines which have been considered so original.

Our author bears testimony to the spirit of true philosophy and research exhibited in Dr. Parry's works—"which has been equalled by few of his cotemporaries, and has rarely been surpassed, in medicine at any period." By this tribute our author does not bind himself to a tame acquiescence in all Dr. Parry's tenets, some of which appear to him essentially erroneous. But notwithstanding this defect in some of Dr. Parry's doctrines and principles—"they are nevertheless illustrated by so extensive an experience, and by observation of so much worth, that he who is not acquainted with the results of this experience and observation, will fall short of the most enlightened views of medicine which are furnished by its present state." Our readers are too well acquainted with Dr. Parry's pathology to require any recapitulation of his doctrines here; and as the greater part of Dr. Pring's critique turns on the action of the arteries in health and disease—a subject on which we have often enlarged in this Journal, we find little in this chapter on which we can animadvert with advantage to our readers. Some points, however, we shall cursorily notice. We stated in our review of Dr. Good's work, in the last number of this Journal, our disbelief of a phenomenon said to be observed in the arteries of an inflamed part—namely, a greater number of pulsations than in the arteries of a sound part in the same subject. Eight years ago our author was a believer in this doctrine; but since that period he has become sceptical, and inclines to think the whole a delusion. We are confident, from considerable attention to the subject, that the existence of the phenomenon in question is a physiological impossibility.

"From having repeated this kind of experiment many times, I am inclined to think the result of a general examination of the fact in question, of the same kind, would be a decision in favour of the equal pulsations of the arteries of inflamed and of uninflamed seats: in other words, that both correspond with the action of the heart; and consequently, that a proof of an independent faculty of contraction and dilatation belonging to the arteries is not to be derived from this source." 117.

In respect to the litigated question, whether or not the arteries *alternately* dilate and contract, in accordance with, and, in fact, producing the pulse, Dr. Pring seems to take, with ourselves, the negative side of the question, though with some hesitation. "I incline to the opinion," says he, "that the proofs are stronger against than in favour of the alternate dilatation and contraction of arteries." And again, a little farther on, he observes—"If it is proved, as upon the whole I think it is, that the arteries have no mechanical action by which they can contribute to the circulation, this phenomenon, in the present stage of our physiological progress, remains absolutely unexplained." It is inexplicable on mechanical and chemical principles, he avers, and therefore we must have recourse to vital.

"Little more is at present confessed, with respect to the arteries, than that they are passive tubes, endowed merely with a vital, or tonic, and an elastic power of contraction.

"But it is probable that the different orders of vessels have powers which are relative to the fluids they circulate, or to those exposed to their action, which are additional to those of mere contractility. That their vital properties are capable of elaborating changes on their fluid contents, and of producing many phenomena which occur in the course of the circulation, and which certainly cannot be explained in agreement with any mechanical laws." 121.

Something equivalent, Dr. P. remarks, to the agency in question, is tacitly confessed with respect to the secerning extremities of arteries—a power superadded to, and apparently independent of mechanical agency. We see also the mesenteric absorbents exercising a faculty of selection or separation, by which they uniformly obtain a similar fluid from amidst the heterogenous mass with which men, to gratify their palates, take the liberty of filling their bowels. The separation and conveyance of this fluid from the intestines to the subclavian vein is not to be explained without a recurrence to the agency of these same mysterious properties of life. Any inquiry into the identical nature of these living properties would be labour lost, and therefore we can only seek for some analogical explanation. We are reduced, says our author, to two alternatives—"1st, That the vital properties of the vessels, by an immediate influence upon the blood contained in them, assist in the transmission of this fluid throughout their respective systems: or, 2d, That this end is promoted by a function of the capillaries." How the first supposition can be maintained, without the mechanical agency of contraction and dilatation, we are utterly at a loss to conceive. The second alternative—the agency of the capillaries—may be of a less visionary character, Dr. Pring thinks. We agree with him that the capillary vessels are endowed with various and wonderful powers to accomplish their different tasks; but when he imagines a power of *attraction*, which they possess for the purpose merely of assisting the heart in the *motion* of the blood, we leave him to his imaginings, as we consider the capillaries have other fish to fry. To us it appears most astonishing that men, after seeing a powerful muscular apparatus placed in the centre of the vascular system, pumping the blood from the veins, and propelling it with great force along the arteries, should yet ransack their brains for auxiliaries to this muscle, among capillaries that are acknowledged to have other and distinct operations of great importance to perform! If the capillaries are so kind as to assist the heart in moving the mass of blood, why should not the heart, in return, assist the capillaries by secreting a little of the bile, or the urine, or the perspiration? Really we think it a very ungrateful organ not to repay the manifold obligations it is under to the capillaries for thus voluntarily doing the duty of the heart in addition to their own!

"It has been remarked," says Dr. P. "by Haller, that the circulation is continued in the small arteries after the application of a ligature to the aorta, or even after the heart has been removed; a circumstance which agrees perfectly with the theory here suggested, although it has not, to my knowledge, been hitherto explained." 127.

Why this has been long and perfectly explained by Dr. Parry and others, who have shewn that the arteries, in a state of health, are always distended by blood, beyond the calibre to which their elastic and vital powers would reduce them, were the supply of blood from the heart cut off. When a ligature is placed on the aorta this supply is interrupted, and the arterial trunks go on contracting and pushing the blood through their corresponding capillaries till, in fact, they become completely emptied, as we see after death. This is plain and obvious to the meanest capacity—but on Dr. Pring's *too acute* perception it is lost. Thus a "microscopic eye" will enable a man sometimes to see minute objects that escape the optics of others; but, on the other hand, it often prevents him from seeing the most sensible phenomena that pass before him.

But while we reject this hypothesis of Dr. Pring's, respecting the magnetic or *attractive* power of the capillaries, in *aid of the heart,* we are far from denying them this power *for other and more necessary purposes.* We firmly believe, that when the *nerves* of a part are excited, whether by internal or external causes, they communicate a power to the *vessels* of that part of attracting a larger share of blood than they usually contain. This is what we have often alluded to as an erythism or erectile process or power of the vessels. We have a striking example in the vessels of the membrum virile, which suddenly expand themselves, as it were, when certain objects strike the mind's or the body's eye. And we every day see the same thing in other parts of the body, when lytta, for instance, is applied to the skin. The nerves are first excited, and then the capillaries fill with blood.*

* It is certain that the heart has the power of expansion, and by

So, when any stimulus is applied, or wound inflicted, we witness the same phenomena. No doubt this power is a wise and important gift, and designed to answer most necessary purposes in the system; but we cannot bring ourselves to believe that it is exerted at every systole or diastole of the heart, to aid that organ in the mere circulation of the blood —an aid which seems by no means necessary.

Nec deus intersit nisi dignus vindiæ nodus.

Dr. Pring has taken up more than a score of pages to bring forth the conjecture, for it admits not of direct proof, that determinations of blood are not the first link in the chain of diseases, whether local or general, but that, on the contrary, the nervous or sentient system of the part is first disturbed or excited, and then follows the vascular impulse. We appeal to our readers whether this be not the doctrine we have uniformly maintained for years past, and therefore we need not go over the same ground again.

While our author denies that determinations of blood are invariably causes or concomitants of disease, he acknowledges that they are so generally. Yet he thinks that blood-letting is too implicitly trusted to in the treatment of inflammatory complaints.

"I have heard," says he, "of persons losing a hundred ounces of blood in twenty-four or thirty hours in pneumonia, and sometimes of their having dropsy shortly afterwards. I presume, as I have met with many cases of pneumonia, and some of them very severe, that if this violent practice had been necessary, I should by this time have had reason to think so. I do not remember having had occasion to take above sixty or seventy ounces of blood from a patient in pneumonia, throughout the course of the disease: I have rarely found it necessary to take above thirty: and yet, negligent as this might appear of an important measure, an uniform success has proved to me, that copious blood-letting is not so essensial as it has commonly been considered. Principally with a view of ascertaining the powers of other remedies, or how far they may stand in the place of bleeding, I have treated a case of pneumonia successfully without a single depletion of this kind. The patient took first a strong purgative of calomel, salts, senna, and jalap; a state of constant nausea, with sometimes vomiting, was afterwards maintained by means of squill, emetic tartar, ipecacuanha, and nitre in large doses, with frequent

---

analogy it is probable that the arteries may have the same; yet it is evident that the mere *relaxation* of tonic power in any set of vessels would cause them to swell with blood, as there is a constant pressure on this fluid, which would force it in excess to whatever point there was least resistance. But in whatever way this is done, there can be little doubt it is through the agency of the nervous system.

purgatives of aloes, calomel, salts, senna, jalap, &c. and the chest was once blistered. I have not frequently met with symptoms more severe than those of this case in the beginning; and a case never occurred to me, the progress of which was more favourable. The means just mentioned gave me almost an absolute control over the symptoms: and with those who understand their principle and employment, there are few medical agents more powerful. It is possible, by these means, in conjunction probably with a bleeding, to make a very decided impression upon an inflammatory disease; sometimes to produce syncope in a few hours after their institution: and there are few cases in which this co-operation of bleeding, blistering, purgative, nauseating, or even emetic medicines, all producing their effects upon the system at the same time, is more strikingly advantageous than in those of bronchitis, or of croup.

"I have known pneumonia, in about the middle of its course, when the feet and legs became red and anasarcous, with a pulse of a hundred and thirty in a minute, treated by a diet of beef steaks and raw onions, a quart of porter, and two or three glasses of gin and water per diem, with scarcely an aperient once in three days. This patient recovered in a short time: the treatment was instituted by a very old practitioner, whose experience had been so extensive, that I know of few men in the profession who could have had so much. On mentioning this case to the late Dr. Parry, he said, he once knew a man who fell from a scaffolding many feet from the ground, and when people expected that all his bones were broken, he got up, to their surprise, and walked away unhurt: but, Dr. Parry remarked, this does not prove that persons might fall from scaffoldings twenty or thirty feet from the ground with impunity. Few men have had a more extensive experience than the late Dr. Parry: but that of the gentleman who instituted the above stimulating plan was much greater; and he, in his turn, would consider recovery from anasarca, even though connected with visceral inflammation, by any but stimulating means, as complete an anomaly as the accidental integrity of the mans's bones who fell from a scaffolding thirty feet high." 207.

Nevertheless our author is far from objecting to bloodletting in pneumonia. He considers it, indeed, as the main item in the methodus medendi.

"During the winter of 1820 and 1821 bronchitis prevailed as an epidemic; and many of the cases, after the first inflammatory violence of the symptoms, was subdued by bleedings, blisterings, and all the artillery of the antiphlogistic plan, ran a long course, which threatened to end in consumption. The chronic symptoms were cough, short breathing, pain, or cough, or both, on inspiration, moist and sometimes clean tongue, pulse averaging from ninety to a hundred or a hundred and ten, flushing of the face, heat of skin, and buffed blood. In some of these cases, in which these symptoms did not yield to the usual remedies, and their fatal termination appeared almost inevitable, I have given two grains of calomel, with

James's powder, three times a day. I had not occasion to resort to this practice in more than four or five cases, and they all recovered. The calomel quickened the pulse a little before the gums were affected, but after a slight ptyalism was produced, the pulse commonly subsided to seventy; the patients lost their other symptoms, and were regularly convalescent from that period. I have succeeded also by suddenly mercurializing the system, after bleeding, blistering, and a purgative have been premised, in bronchitis, which, from having been previously neglected, has, in an advanced stage, threatened suffocation every hour. In order to make the greater impression on this state of disease, I have combined emetic tartar with the mercury, as by giving two grains of emetic tartar, and ten of calomel, every six or eight hours. These medicines, in this combination, are capable of producing their separate effects." 209.

Our author has tried mercury to the extent of salivation (which, in general, is not easily produced) in phthisis; and, in several cases, all the symptoms of the tubercular form of the disease were suspended for a time by the remedy. The recovery has been so considerable, in some instances, that the patients were pronounced well by their friends; but, as may be expected, the suspension of the disease was only temporary.

"I have, on the other hand, known cases of ulceration, following acute inflammation of the lungs, which has become chronic, and continued many weeks with quick pulse, colliquative sweats, pain on inspiration, buffed blood, and purulent expectoration, which have recovered perfectly after salivation." 210.

We have often proved the truth of the following observation, which we deem of some importance.

"But, as before remarked, it is sometimes extremely difficult (I have once found it impossible) in these, as well as in some other forms of organic disease, to make mercury produce its usual effects. It will quicken the circulation: but the local determination, which is in general connected with every preternatural diathesis, will oftentimes be to the seat of the disease, rather than to the glandular system, on which mercury, at other times, appears to exert so specific an operation. The symptoms of the disease, under such mercurial influence, are in these cases rather increased: and the vascular action excited is unfavourable to the local disease, or rather concurs with the particular effects of mercury on its seat, to hasten its progress." 211.

Dr. Pring's experience is decidedly against blood-letting in any form of phthisis, and to him setons, issues, and perpetual blisters, have generally appeared rather prejudicial than otherwise. On these last points, his experience and ours do not exactly coincide. Our readers will remember some observations which we made lately on the danger of large and repeated depletions in acute rheumatism. We are glad to find that Dr. Pring's experience corroborates what we have there said. Our author trusts more to purgatives with calomel, elaterium, aloes, salts, senna, &c. We deem the following short case worthy of being inserted here, as we have witnessed several such, and with similar results.

"A gentleman had chronic rheumatism, chiefly affecting his knees and shoulders. He went out on a cold, damp day: in the evening he had rigors; the rheumatic pains left the extremities, and he was taken with something like syncope, sense of constriction at the bottom of the throat, of weight on the chest, with a fluttering irregular pulse of about a hundred and sixty in a minute. I opened a vein in the arm, but could not get more than four or five ounces of blood, which was buffed. I gave him a full dose of calomel, salts, senna, and jalap, which produced eight or ten stools in as many hours, covered his chest with a blister, &c. The next day he was able to lie flat in his bed, and his pulse was more regular and slower, though still quick and intermittent. He took repeated doses of squill and calomel, with salts, senna, &c. every morning. The squill sometimes produced vomiting, and in about four days his mouth became affected by the calomel; his pulse almost immediately afterwards came down to sixty, was perfectly regular, and all the other symptoms ceased. He was confined a few days to the house by the soreness of his gums and tongue; this affection subsiding, he recovered rapidly." 217.

He has "found chronic rheumatism, in other instances of considerable duration, yield, after bleedings had been premised, to alterative doses of calomel with sarsaparilla. The gums have commonly been a little affected before the symptoms ceased, and the alvine evacuations have been copious, both during the course, and after it, without any additional purgative." Colchicum has not succeeded in our author's hands. We have often found it beneficial, especially when it purged or vomited. In a few cases the extract of the seeds of colchicum (one grain for a dose at bed time) has proved more successful than any other preparation of the meadow saffron. The following passage corroborates the strictures which we made some time ago on Dr. Abercrombie's therapeutical indications in abdominal inflammations.

"In peritonitis, I have trusted also much more to purgatives than to bleeding, and I have no reason to regret this confidence; for out of many cases, under every variety of circumstance, I never met with one which terminated otherwise than favourably. It has appeared to me the first object in inflammations of the bowels, whichever coat may be the seat of it, (and on this point our diagnosis is imperfect) to overcome the constipation with which such inflammatory disease is commonly, or frequently attended. This design is chiefly founded upon the experience that, when this effect is accomplished, the cure is in our own hands. The theoretical design of this practice is less to evacuate the bowels, on the supposition that their contents may be instrumental to the disease, than to subvert the state of disease in the bowels, by the employment of measures which act upon its seat. Among other modes of the operation of purgatives in inflammation of the bowels, perhaps the secretion which these means excite throughout the intestinal canal, is not the least important. We know that secretion is an ending of inflammation, and that it is frequently the spontaneous mode of relief to the vessels of an inflamed seat.

"The use of purgatives in enteritis has been objected to, on the ground that they act by stimulation; and that stimuli, applied to its seat, must increase inflammation. The effect, however, is otherwise; and the testimony of experience must on this, as on other occasions, supersede all *a priori* reasoning. But, as a matter of reasoning, perhaps the conclusion against purgatives on this ground is not legitimately inferred. It does not follow, that an agent which is related with a secreting function, so as to increase it, should also be so related with inflammation (which frequently suspends secretion,) as to augment its intensity. On the contrary, in the way of reasoning, it would appear, that if secretion is suspended by inflammation, that which restores secretion must diminish inflammation. Setting reasoning, for the present, aside, I suspect, that in the cases in which purgatives have been supposed to increase intestinal inflammation, it is because these means were inadequately employed." 219.

Purgatives, in intestinal inflammation, have been objected to on the ground that they are quickly rejected by vomiting; but this objection is not valid. Our author, after premising bleeding, gives at first six or eight grains of calomel, followed in an hour by a black draught, with half a drachm of jalap. If this be rejected by vomiting, it is repeated in an hour or two, and so on, with this or other forms of purgatives, until the bowels are opened, when, in general, we find the ball at our own foot. Injections are powerful auxiliaries to these means.

"Among other instances of intestinal inflammation, I have met with many of puerperal peritonitis. They have invariably done well under adequate purging: bleeding has, in some instances, been premised, but never carried to a great extent; and of this disease, as of some before mentioned, I may say, the greatest number of fatal instances which have come to my knowledge have been those in which the treatment has been rested upon bleeding, to the neglect of other measures. Blisters on the abdomen are useful auxiliaries, if the irritability of the system will permit their employment. Leeches may also be beneficial; but both these means are, I believe, chiefly useful, inasmuch as they furnish an excuse for covering the abdomen with a large bread and water poultice.

"Although I have never myself employed spirit of turpentine in puerperal peritonitis, I have had opportunities of witnessing the effects of this practice; and the results, so far as they have been known to me, have been favourable. The mode in which it has been given has generally been in doses of two drachms or half an ounce of the turpentine, with an equal quantity of castor oil; and either of these doses has been repeated, perhaps, in six hours. The mode of operation of this remedy has appeared to me to be the common one of purgatives; of which class of medicines it is one variety." 221.

Although our author has been generally successful by the means abovementioned, yet cases have occurred where he was foiled, and the disease seemed hastening to a fatal termination. In such instances he has given, as a *dernier ressort*, ten grains of calomel every six or eight hours, and with success. "Copious stools have quickly followed a sudden salivation, and a favourable convalescence has afterwards been maintained by purgatives of the weaker sort."

"I know of no case in which this remedy, pushed to such an extent, is more indicated by principle than in this: the general determination to the glandular system before spoken of, is one among other modes of subverting inflammation in a seat of disease, and the importance of resorting to it is great, where the continuance of inflammation may be fatal by its relation with the function of the structures which it invades, or by the prejudice which, in other instances, may be suffered from disorganization. On this latter ground, the practice has been adopted with a success to which my own experience bears concurrent testimony in iritis, the progress of which I have found immediately arrested as soon as the saliva began to flow. I have also, in children, known constipations which have resisted all previous means give way when the mouth has become affected by calomel." 222.

In phrenitis, from local injury, our author acknowledges that the superiority of blood-letting over all other means is very conspicuous. Purgatives and nauseating antimonials, however, are important auxiliaries. We have had occasion often to allude to the curious and very interesting experiments of the late Dr. Thomas Seeds, who shewed that by bleeding animals to death the vessels of the brain were gorged, and sometimes the blood, or other fluid, extravasated. The following case will be found to bear on this curious result of extreme bleeding, besides affording some interesting therapeutical information.

"A woman was delivered suddenly and unexpectedly, whilst standing in her room, at a distance from the bed; the child fell on the floor, but the funis was not broken; there came on instantly a copious flooding. I found her, as soon as I arrived, insensible, and the floor was deluged with blood: she had no pulse at either wrist, &c. Twelve hours afterwards she had a pulse of a hundred and twenty in a minute, bounding of the carotids, dilated pupils, and complete insensibility. She lay in this state more than a week. I purged her every day; her pulse was always reduced after the action of the purgative, and regularly increased during any long interval of its operation. This woman was a catholic; and at the end of a week, as it was expected she was to die, she received extreme unction, and every thing was made ready for this event. I continued the purgative of calomel every night, and salts, senna, and jalap, the following morning. On the ninth day she recognized her husband and children, spoke to them, &c. and, as she was better, I thought I would omit the purgative: she had no stool for about twenty-four hours, and then relapsed into her former state of insensibility. I gave her an active purgative, and she recovered her faculties and speech, in as great a degree as before the omission of the purgative, as soon as it had operated. Her convalescence from this time was regular, and during the whole period of it, a slight purgative, frequently repeated, together with a gradual repletion, kept her free from any considerable degree of affection of the head." 224.

Some remarks are made on the treatment of insanity, apoplexy, &c. Dr. Pring relates the case of a female, who became insane when she got thin, and got sane again on becoming corpulent. "The cure of insanity," says he, "by obesity, is remarked as a common circumstance. It may be cured also by an abscess, by a carbuncle, or by pregnancy; facts which, if duly considered, *are not very complimentary to human genius*." We really see no great reason to depreciate human genius, (which cannot be manifested without a sound brain) because certain changes in the constitution render that brain capable or incapable of such manifestation. But we do think that it is "not very complimentary to human genius," to be perpetually catching at every weakness and imperfection in our frail nature, for arguments in favour of levelling man with the grass he treads on. Yet the Materialist is eternally intruding his own sublimity of genius upon us at the expense of that human nature in which he seems to forget that he shares in common with the rest of mankind.

Our author is disposed to think that blood-letting in apoplexy is in some, though not in all, cases, overrated. As a specimen of Dr. Pring's mode of objecting to received principles in pathology and therapeutics, we may instance his reason for the above doubt. As blood-letting, says he, is almost invariably resorted to in apoplexy, our experience of its efficacy is, on this account, defective, because we have not the opposite experience for comparison, viz. total neglect of bleeding. But have we not strong evidence when we see a patient recover his sensibility in exact ratio as we draw blood from the system—and when we find, in fatal cases, effusion of blood in the brain, or extreme turgescence of its vessels? That blood-letting will not save life in all cases is most true. Who could expect such a thing, who knows the various states and degrees of disease in the brain, or who sees the quantum of blood sometimes effused into the substance or on the surface of that organ? But no species of evidence is free from doubt, cavil, and objection in Dr. Pring's mind. We verily believe that he entertains very serious doubts of his own existence—at least we are sure that he could start objections to every proof that might be offered in the affirmative. Now, while we are advocates for receiving medical evidence with proper caution, and for guarding against hasty and sweeping conclusions, we are, on the other hand, convinced that pathology and therapeutics will not be advanced by this eternal spirit of cavil and scepticism which is pervading some of the rising generation of medical philosophers. As we have often before observed, mathematical demonstration and logical precision are not to be expected, where the laws of life and organization are concerned. We must therefore be often contented with probability instead of certainty—with conjecture (founded on such evidence as we can collect) instead of proof. And yet we see these ultra-sceptics sometimes grounding *their* dogmas on very insubstantial facts. Who would expect such a case as the following gravely presented to men of experience at the bed-side, as a proof that the good effects of blood-letting in apoplexy are greatly overrated?

"A gentleman of a full habit, between fifty and sixty years of age, had apoplexy after dinner; he was bled, and recovered his speech in a few hours, but had lost the use of one side: his pulse was a hundred and twenty. He was bled the same day from the arm, and fifty ounces of blood were taken from the temporal artery: he was bled again the next day, and cupped in the evening. During the whole of this time, from the recovery of his speech, he recognised his friends, and talked with very little incoherence. The bleeding had chiefly for its object the reduction of the pulse: in this intention it failed totally. He lost about a hundred and thirty ounces of blood in the first two days, and on the third he died. This patient I saw only by accident, or from locality, once or twice." 230.

Here we have evidence that bleeding *relieved* the symptoms, and roused the patient from insensibility to a recognizance of his friends, and to coherence of speech. No dissection is given; but little doubt can be entertained that irreparable mischief was produced in the brain by the extravasation of blood, which would there have been found.

We do not contend, indeed, that bleeding will cure disease in the vessels or disorganization in the structure of the brain —circumstances which often lead to apoplexy. But we contend that when the effusion or the congestion actually takes place, we have no remedy equal to bloodletting in obviating the effects of such extravasation or turgescence, which we maintain to be the *immediate* cause of the apoplectic symptoms.

We agree with our author in censuring the too frequent detraction of blood from the system as a preventive or cure of those determinations which we see take place towards particular organs. In such cases we would only bleed to obviate urgent symptoms, and trust to spare diet and revulsive irritations for the rest.

Our author considers epilepsy as nearly allied to apoplexy —and that it may, in general, be prevented by occasional bleeding and great temperance in diet. But if the disposition be accompanied by symptoms of chronic affection of the head, these symptoms will not be mitigated, but often increased, by a course of depletion instituted for their cure.

"Chorea, which in its pathology is probably allied to epilepsy, I have no reason to think much benefited by blood-letting. In the few cases which have occurred to me of this disease, some of which were severe ones, it has yielded to purgatives in about three weeks. The purgatives were chiefly combined ones of calomel, aloes, scammony, salts, senna, jalap, and rhubarb, given so as to procure three, four, or half a dozen stools every day: the stools have commonly assumed a healthy appearance towards the end of the above period, and the spasmodic action of the muscles has quickly ceased." 245.

Dr. Pring remarks that bleeding, in nervous disorders of the head, is sometimes indispensible as a measure of relief; but if frequently repeated, that the irritability of the heart is generally increased, as well as the local affection of the head. Patients of this description, he avers, enjoy the best health when they are brought to bear a full diet.

"The most common symptoms of nervous disorder of the head are pain, throbbing, sense of tightness across the forehead, rather white, but moist tongue, and the pulse, during an exacerbation, between ninety and a hundred; at other times, perhaps, between seventy and eighty. What the precise seat of the pain might be is, in many of these cases, doubtful. I have often thought it to be in the scalp, from the great soreness felt in this part after the pain has ceased; and also from the circumstance, that a handkerchief bound for half an hour so tightly round the head, as to intercept, or diminish the flow of blood to the scalp, will often afford a temporary cure. In what proportion of cases this seat may be supposed, or by what circumstances it may be distinguished, I do not at present possess facts enough to determine. It is, however, to be remarked that, on the application of such pressure, a sense of bursting is felt in the vascular system of the head; each systole of the heart distends sensibly the vessels on the side of the pressure which is nearest the heart: this sense of distention commonly abates in five or ten minutes; and perhaps at the expiration of half an hour, on withdrawing the handkerchief, the head feels almost benumbed, and afterwards hot, but without pain. The practice of preventing the circulation in the scalp in this way may, perhaps, not always be free from hazard, if the seat of the disorder is within the cranium." 247.

The following case exhibits a striking instance of what we might term a vacillation of the circulation, or, perhaps more properly, an oscillatory disposition to vascular excitement.

" A man of rather a full habit, and pale complexion, about forty years of age, by trade a baker, was attacked by violent pain in the head. He was bled repeatedly from the arm, from the temporal artery, by cupping, by leeches, took purgatives, was blistered, &c. This treatment, which was continued five or six weeks, scarcely produced any mitigation of the symptoms: in the course of it, and towards the end of the above period, he had a copious spitting of blood, which was sometimes frothy, was coughed up, and evidently proceeded from the lungs. As soon as this hemorrhage occurred, the head, which had not been relieved in any degree by previous blood-letting, continued up to this period, became perfectly free from pain. The expectoration of blood continued a few days; it ceased; and the pain returned in the head with unabated violence. For some weeks afterwards, the seats of affection alternated with almost perfect regularity; one day, pain in the head; the next, hæmoptysis. The disorder of the head eventually prevailed; the hemorrhage from the lungs ceased; the man became mad; he was sent to a lunatic asylum, and died of apoplexy. This case, in connexion with some preceding ones, shows forcibly how inadequate blood-letting is for the prevention of disease, of which determination of blood is even a conspicuous sign, unless it has a curative relation with the state of disease, upon which the symptom of preternatural determination depends." 253.

In hæmoptysis our author has had reason to think that saline purgatives, with sometimes calomel, nitre, ipecacuan, &c. have been more effectual than repeated bleedings.

" The pathology of hæmoptysis furnishes two important practical distinctions, namely hæmoptysis with, and without buffed blood. If blood, taken from the arm, in cases of hæmoptysis, is buffed, the local disease tends to rapid disorganization; and unless arrested by treatment, terminates speedily in consumption. If, in such cases, the blood is not buffed, there is no immediate danger of consumption; the only danger is from hemorrhage: and this danger is not trifling. The most inconsiderable spitting of blood is always a symptom which requires great attention. If a small vessel of the lungs is ruptured, it proves a disposition in the vessels to this effect; and there is no security, but that a large one may next give way." 256.

"In hæmoptysis with buffed blood the symptoms cannot be too rapidly subdued by antiphlogistic means. Bleedings of sixteen or twenty ounces, calomel, active saline purgatives, full doses of nitre, squill, ipecacuanha, emetic tartar, &c. and a diet of cold gruel, tea, barley water, &c. should be employed in the beginning, together with perfect repose. If the case becomes chronic, small bleedings, as a collateral treatment, are advantageous, together with nitre, and constant purging by Epsom salts: I have ample reason for thinking the repetition of large bleedings prejudicial; and a treatment, in which blood-letting is made the only remedy, altogether inadequate. I have known all the symptoms cease, in these cases, and the blood has no longer exhibited fibrine, when the calomel, which was given in doses of three grains every night, followed by salts and senna the next morning, has affected the gums. I have known also the coagulation of the crassamentum gradually weakened, and the exhibition of fibrine cease, under a treatment by a grain of acetate of lead three times a day, in combination with a full dose of squill, and a small one of digitalis; the saline purgatives, and occasionally calomel, have also been employed at the same time." 257.

In our review of Dr. Good's work, (page 817 of our last volume,) we stated that we had seen a few cases where the pulsations of the heart exceeded those at the wrist, being generally double the number; but that we never saw an instance where the arterial exceeded the cardiac pulsations in number —in short, that we considered the latter a physiological impossibility. The following cases hews that Dr. Pring's experience is in accordance with our own.

"The patient was about forty years of age; he had suffered in the course of his life two or three attacks of acute rheumatism, which showed always a great disposition to metastasis, and his heart was more or less affected in each attack. About two years since he had acute rheumatism, affecting chiefly the arms and legs; during this attack he was under my care. In the course of the disease the swelling of the hands sometimes ceased suddenly, and was succeeded by violent pain in the heart, sense of weight on the chest, and of suffocation, with a quick and intermittent pulse. Towards the termination of the case, the stroke of the heart was precisely double that of the pulse at the wrist. When the pulse beat fifty, the heart beat exactly a hundred strokes in a minute! when the heart beat eighty, the pulse was forty. The case was chronic, and my examinations frequent, and the result was always the same: the pulse of the heart was invariably, to a single stroke, double that at the wrist; and there was in general no very perceptible difference in point of strength between the beats of the heart; or if, at any time, a difference could be felt, it appeared as if the second stroke was rather the weaker of the two." 266.

With Dr. Pring's attempt at explaining this phenomenon we shall not trouble the reader, as it is far from satisfactory.

We agree with our author that hydrothorax and hydropericardium are less successfully treated by bleeding than by purgatives—and that the effects of the latter in relieving patients from the pressure of fluid on the heart, lungs, and diaphragm, are often surprising.

"A woman, about sixty years of age, had hydrothorax, connected with disease of the heart, for many months. When I first saw her, she was sitting upright in her bed; her breathing was exceedingly laboured, her pulse intermittent, and only thirty-six in a minute. Ascites had of late been added to hydrothorax, and I found her legs tightly bandaged to prevent their swelling. The woman appeared almost in articulo mortis: the bandages were of course removed. I covered her chest with a blister, and gave her a full dose of calomel, squill, aloes, and elaterium, followed by a black draught with jalap. The alvine evacuations were, in consequence, very copious; the kidnies afterwards acted inordinately, and she passed many pints of almost limpid urine every day. In less than a week, under repetitions of the same medicines, she was totally free from all her symptoms. The plan was continued in less degree, by way of prevention; but she was well enough to walk out, and became tired of discipline." 269.

She was two or three times relieved in this way afterwards, but ultimately died comatose, having refused to submit to medical discipline. We have met with several cases exemplifying the above.

Idiopathic dropsy, in our author's experience, has generally yielded to purgatives, as of calomel, elaterium, gamboge, with large doses of squills and small ones of digitalis. "It most commonly happens that some degree of ptyalism is produced by the calomel before the disease is completely emoved."

"The employment of mercury in dropsy has been an empirical practice; but its powers deserve all the attention which observation and science can bestow. I believe mercury will very frequently cure the disease, if this remedy is carried to a great extent. But it is often difficult in this, and other local diseases of a fixed character, to make mercury produce its usual effects; and upon its producing salivation, I have reason to think its curative operation depends. Not that the mere salivation is of any use; but it is an index of a more general affection; and I have never met with a case of dropsy, unaccompanied by disease of the heart, which did not cease under a salivation, continued for a sufficient length of time. It has been proposed to limit the use of mercury to those cases of dropsy only which are not inflammatory. I have used it with the most perfect success, where the blood has been buffed; and I have certainly found a treatment by bleeding fail, although the blood always exhibited a surface of fibrine. But my experience on this point has not been sufficiently extensive to decide so important a practical question." 271.

As dropsy is generally symptomatic of disease in the heart or the liver, it is reasonable to suppose that, in the latter case, mercury is the only medicine that is likely to prove efficacious. Dr. Pring properly observes that the cure of symptomatic dropsy, as connected with liver disease, is no trifling illustration of the powers of medicine—while it may be observed that the medicines which will accomplish this end are of no very gentle kind. The following case, besides exemplifying the foregoing remark, is very instructive in itself, and is one of those which often tend to establish a man's reputation even at an early period of his professional career.

" A woman, in other respects of a good constitution, between forty and fifty years of age, suffered occasionally from chronic inflammation of the liver. This viscus was, at these times, enlarged, indurated, and painful, on pressure: she had three or four of these attacks in the course of two years, and they yielded to the ordinary treatment in a fortnight, three, or six weeks; and towards their termination, the stools were sometimes copious, and almost black. She was subject to more frequent attacks of bilious vomiting and purging, which commonly ceased after one or two doses of calomel, followed by a cathartic draught. During the above period she suffered only occasional inconvenience, and very little confinement from her complaint. The liver again became enlarged and indurated; the disease did not, at this time, yield to ordinary remedies: she was purged, bled, leeched, cupped, and blistered, repeatedly; the tumor in the side continued to increase, her legs became anasarcous, and fluctuation was perceptible in the abdomen. She took principally purgatives, and the topical treatment of the side consisted of local bleedings and blistering: she took small doses of calomel, as a grain, three times a day, and rubbed in mercurial ointment upon the side. This plan was continued nearly three weeks, and the quantity of calomel, during this time, was increased to six or eight grains a day. The gums were not, however, in any degree affected; the enlargement of the liver continued to increase; the anasarca of the legs extended from the feet to the hips, or to the sides of the abdomen: her hands and arms were also anasarcous in a slighter degree; and her respiration was difficult, owing in part to the tumor of the liver, and in part to fluid in the abdomen. Her case was considered a hopeless one; and from experience of the inefficacy of the remedies hitherto employed, it was abandoned, except by myself. I told her there was still a chance left for her; that I was not very sanguine of the result; but that I would give her this chance if she chose to submit to the discipline. Her death seemed otherwise inevitable, and she therefore consented.

" As the former doses of mercury had not produced the effect upon which I believed the curative action of this remedy to depend, I determined to produce this effect at all events; and as there appeared no time to be lost, I gave her ten grains of calomel every night and morning, and directed her to proceed with the mercurial frictions. She continued these doses about a week; the stools, consisting of very depraved secretions, became copious without any other purgative, and her gums were swollen and tender. I then gave her eight grains of calomel, with squill, every night and morning, and she continued these doses about a fortnight. She occasionally took also a black draught, with half a dram of jalap in it. Her gums, during this fortnight, continued much swollen; the palate also was thickened; there was a little increase of salivary secretion, but no ulceration of the gums or tongue. She had every day copious stools, consisting of vitiated secretions of apparently every variety, black, green, &c. with a considerable proportion of mucus, and sometimes a very little blood. The tumor in the side had nearly disappeared; she could lie flat on her bed; there was very little enlargement of the abdomen, but the legs continued anasarcous. She afterwards took every night pills, with five grains of calomel, three or four of gamboge, half a grain of elaterium, and three grains of aloes, followed every morning by a black draught, with half a dram of jalap in it. This plan was scarcely interrupted for three weeks; and when it was, it was only to omit the purgative, and resume, for perhaps a couple of days, the ten grain doses of calomel, when the symptoms under the purgative showed a disposition to increase. Her pulse throughout this course was, upon an average, between 90 and 100. She also occasionally took an emetic; and sometimes, by way of varying the purgative, a dose of spirit of turpentine, with castor oil. The evacuations from the bowels were still exceedingly copious, and consisted chiefly of the secretions before described. Her diet was of the most nutritious quality. As a compensation for this severe discipline, I allowed her to gratify her palate by eating and drinking as her fancy dictated, without any restraint. My real motive for this indulgence was one founded in an experience of the advantage, in many chronic cases, of sustaining with one hand, while we are pulling down with the other. The curative design is to subvert a state of disease: and if the disease is a formidable one, and this design is really to be accomplished by medicines, without being indebted to Nature, (who is in such instances driven towards our purposes very much against her will,) the means by which this can be accomplished must be of no trifling powers. This discipline was continued about two months, with but little interruption; at the end of which time, as no vestige remained of her complaints, it was presumed that the remedies had subverted the diseased, and were followed by the resumption of a healthy state of the functions. I did not, however, choose to trust over much to this presumption, knowing that it is one thing to cure symptoms, and another to subvert the tendency by which they were produced, and may be produced again, when the influence of the remedy has passed. She therefore continued to take her pills, with calomel, gamboge, elaterium, &c. together with the draught, containing half a dram of jalap, almost every night and morning, for two or three weeks longer: these doses were then given every second day, every third day, and irregularly three times a week, and twice and once a week for some times afterwards." 276.

This patient's recovery was perfect, and does great credit to Dr. Pring.

Our author hazards some ingenious speculations on the remarkable influence which mercury exerts on the hepatic system in particular, and on the other structures of the body in general; and as there is some novelty, and, we apprehend, truth, in these speculations, we are tempted to give the passage in his own words.

"I do not remember to have ever seen the principle defined on which mercury acts so beneficially in diseases of the liver. If absorbent glands are enlarged, the mercurial diathesis frequently makes them suppurate; but if this does not happen before salivation takes place, or if salivation succeeds to the suppuration of such glands, as those of the axilla, for instance; in the former case, the revulsion made under ptyalism to the secerning glandular system will be followed by a reduction, or perhaps disappearance of those glands which were enlarged; and in the latter, the same mode of operation will frequently produce the absorption of matter which was formed under mercurial influence, but previously to the ptyalism. The mercurial diathesis would also, in many cases, excite suppuration of the *liver*, when this was diseased, as of other structures; but the determination to this gland, produced by mercury, instead of exciting suppuration, forces it upon an active secretion, by which its state of congestion is relieved, as congestion or determination is, in other instances, by a spontaneous, or other ending, in secretion. A quantity of mercury, therefore, which, not producing salivation, would in other structures produce or hasten disorganization, in the liver produces only a less degree of the salutary influence which this medicine exerts upon this viscus when ptyalism, or a general increase of glandular secretion, is excited by it; and if the fixed nature of the disease is a cause why mercury does not produce ptyalism, (as it is in some instances, for the reason that the particular determination of mercury is rather to a previous seat of disease, than to the glandular system,) the local disease is not, in the case of the liver, as in other structures, increased by this particular determination, but still finds, at once a security against the prejudicial operation of mercury, and a vis medicatrix, under its influence, in the secerning function of the gland. Thus, though in diseases of other structures mercury may be prejudicial if it does not produce salivation, although it may cure if it does, every grade of mercurial influence *on the liver* is likely to be advantageous in its diseases; and mercury does not in this, as in other seats, produce opposite effects in different stages of its employment." 278.

The fifth chapter of the work before us is entitled—" Origin of Disease in the Abdominal Viscera." Dr. Pring begins it by observing that it is a prevalent doctrine "that disorder of the stomach or liver is the cause of almost every other local disease." This doctrine certainly is *prevalent* to a certain extent; but surely there are few who adopt it in the latitude stated by our author, what observant practitioner can fail to perceive that the gastric and hepatic functions are daily and hourly disturbed by disorder of other parts, and consequently are only sympathetically or secondarily affected? The long train of passions act on the stomach and liver through the medium of the brain and nerves. Suppressed perspiration will disorder the stomach and liver, the skin being the organ primarily impressed with derangement of function, and so on. In fact, there is no part of the body in which disorder may not originate, and so far from its being occasioned by, may be itself the cause of, the disordered state of the digestive organs. At the same time it is highly probable that the chylopoietic viscera are more frequently disordered, *primarily* or *secondarily*, than any other class of internal organs.

We agree with Dr. Pring in thinking that the greater frequency of gastric and hepatic complaints now than two or three hundred years ago, is far more owing to disturbed function of the brain (which organ is infinitely more exercised now than formerly) than to any depraved or artificial habits in respect to food. Education is pushed farther—refinement is more general—sensibility is more acute than in days of yore. The intellectual and animal functions are improved at the expense of the organic—and hence the great source of chylopoietic derangements.

Dr. Pring takes some pains to classify or arrange the disorders of the digestive apparatus, and exhibit their true relations in disease. He divides them into two classes—the exclusive and the related. The last class is subdivided into a great many orders or genera. Of the exclusive disorders of the digestive organs, our author has not seen more than three or four cases, where they appeared unconnected with other disease. On the other hand, related disorders of the digestive apparatus are very common, as *first*, derangement in the chylopoietic organs producing disease elsewhere by simple extension, in which class Dr. Pring includes some forms of chronic fever, erysipelas, and certain ulcers. *Secondly*, disorder originating in the digestive organs, and ceasing under the occurrence of disease in another seat—cases, however, which, he thinks, are not very common. Thus he has known consumption preceded by severe chronic dispepsia, which last ceased in the course of the former; and so has he seen chronic dyspepsia cease under a cutaneous eruption.

"3. Disorders of the digestive organs originating elsewhere, and exemplifying simple extension of disease, form a very numerous class. To this division belong perhaps the largest proportion of those cases, in which nervous disorder is in connexion with disorder of the digestive organs. This combination of disorder generally originates in habits whose primary relation is with the mind, or the passions; such as habits of business, of study, or of amusements of the anxious and feverish sorts, as those incident to a life of dissipation, the taste for which is sometimes gratified without frequent gastric debauches. If a girl is in love, the disturbance of the brain, by the excitement of a passion, may give her a white tongue, loss of appetite, &c. perhaps also a vicarious determination to the head or chest, connected with irregular menstruation, producing hysteria, or even alienation of mind, or spitting of blood, and perhaps eventually consumption. Such determination to the head, &c. with various shades of modification, are the more likely to occur, if the progress of the amour is marked by systematic opposition, cross accidents, regrets, anxieties; by the alternation of fears, hopes, mortifications, revived expectations, joy, disappointment, &c. These forms of disorder are generally those of simple extension: the function of the brain is first disturbed, the stomach, becomes disordered in consequence; and the disturbed state of the brain, expressing itself with more or less severity in the several instances, continues without mitigation from the secondary affection of the stomach, by which it is more frequently increased than diminished." 297.

4. Dr. Pring observes that disorders of the digestive organs, originating elsewhere, and holding a *curative* relation with the original seat, are of frequent occurrence. Thus, fevers abate on the supervention of diarrhœa—and more especially on the "appearance of stools," "which prove that the *state of disease* is assumed by the abdominal organs of secretion." We cannot perceive the force of Dr. Pring's reasoning, when he concludes that the appearance of feculent matter or stools indicate the transferrence of disease to the digestive organs. We should have come to a diametrically opposite conclusion—namely, that while the motions were watery and diarrhœal, there was really disorder in the digestive apparatus, but that when the motions became feculent, it indicated a return of *healthy function* in those same organs. Doctors, however, will differ in opinion. There are many other cases, indeed, where, under febrile states of the system, a discharge of dark, fetid, and unnatural secretions takes place from the bowels, with reduction of the pyrexia. Even here we can hardly view the diarrhœa as a state of disease in the chylopoietic viscera, but rather a salutary increase of natural function to remove disease from the system.

Our author properly remarks that secondary disorder of the digestive organs, instead of proving curative, often re-acts on, or increases, the primary affection. This state of the digestive apparatus will maintain disorder of the head, and nervous disorder, when the subject has been long removed from those habits or influences which first disturbed the function of the brain, and through this medium the stomach. Thus, also, local injuries first disorder the stomach, and this state of the stomach maintains a disordered state of constitution, in which the local injury participates. Dr. Pring makes some curious observations on dyspepsia, some of which we shall introduce to our readers.

"The most prevalent form of dyspepsia which has occurred in my experience, is that denoted by furred tongue; brackish, or bitter taste in the mouth; irregular appetite, which is sometimes good, but more frequently otherwise; flatulence; torpid bowels; nervous irritability; disturbed sleep, which is sometimes morbidly profound; general uneasy sensation, perhaps with or without pain in the stomach or bowels; occasional depression of spirits; frequent languor; sudden changes of temperature of the skin; cold feet; and most commonly, a tendency to disorder of the head. The degrees and combinations of these symptoms afford the principal diversities which I have observed in dyspepsia." 301.

The plan of restraining the patient from all those kinds of food which are supposed to be prejudicial in this complaint, has never been known to cure a single person in our author's experience. He argues that the stomach can often digest with ease things which physician and patient have thus determined *a priori* it ought not to digest at all. And again, he avers, that, by not keeping the digestive function adequately exercised, it is apt to become capricious, and either loses the inclination or power to perform its duty. And thirdly, that, as the bile is probably the natural stimulus to the bowels, he has reason to think that the food is not a little concerned in enforcing their action—that people may have griping pains, diarrhœa, tenesmus, &c. with white stools, where, of course, there is paucity of bile—that if persons eat sparingly, the bowels will not act regularly for want of adequate stimulus from this source—that if they eat abundantly, the stimulus of the ingesta will, to a great extent, contribute to, or produce regularity of the alvine evacuations.

"I have known a change of diet, from a starving one to one of repletion, followed by regularity of the bowels, so that under the latter regimen the subject did not take an aperient medicine for two years: and under the former, he scarcely ever had an evacuation which was not procured by medicine. I knew a young man, twenty-one years of age, who had dyspepsia; he was, however, in other respects, healthy and vigorous; he had a soft pulse, commonly of sixty, and was able to walk ten or fifteen miles in a day without fatigue. His bowels were very torpid, and he was highly nervous. He had been directed to eat only food of a certain quality, and of this food, which was weighed, to take only a few ounces; to drink water, and to procure stools by large doses of the compound gamboge pill. I recommended him to leave off his pills, eat as much as he could, and of what he liked, and to drink a quart of strong beer every day. He was persuaded to enter, with great timidity, upon this plan; and in three or four days, his trouble was not that his bowels would not act, but that they acted too much, or too frequently. He was frightened at this effect, and returned to his old regimen, which he persevered in; and in a few months he died of an atrophy: my construction of which event was, that he was starved to death; notwithstanding he possessed a good apetite, and the means of procuring as much food as he liked. It is to be remarked that there was not, in this case, any evidence of diseased mesenteric glands." 303.

Although the above doctrines are rather heterodox in medicine, yet we do not deem them entirely fanciful or always dangerous. We think that the physician who recommended the young man such Pythagorean fare and drastic purgatives, was full as wrong as Dr. Pring, who changed the system to alderman's fare and porter. It appears plain to us that the patient's digestive organs were not equal to the task assigned them by Dr. Pring; and that the plenitude of food and porter acted as a purgative, and hurried the ingesta through the bowels. Moreover, we imagine there must have been some serious radical vice in the constitution of a patient who died of atrophy, in a few months, even on the slender fare which was first prescribed for him. Our rule is, to let the dyspeptic patient (where there is no other disease) eat moderately of any food that agrees best with his stomach, limiting the quantity, when sense of fulness after eating, eructations, flatulence, or uneasy sensations obtain. Whether this or the following plan be the more prudent, we leave to the judgment of the reader.—

"Although, in dyspepsia, the bowels may not act with regularity under any plan of diet, I believe their action will approach nearest to regularity under the regimen of repletion, that is, of eating and drinking abundantly: this plan will produce occasional inconvenience; but it is attended, so far as my observation goes, with a better general state of health than when the opposite one is adopted. It will every now and then be productive of an exacerbation of the symptoms; and then a purgative, or a short course of purgatives, with a change of diet will at once be a remedy to the exacerbation, and have the effect of producing afterwards a comparative freedom from the complaint, and an improved state of health. This improvement will, perhaps, last many months; during which time the less the patient thinks about his stomach and bowels the better. Nature might take the matter into her own hands; and if the bowels are neglected, she will, perhaps, give the patient a slight attack of cholera, which will be followed by all the improvement which usually succeeds to a trifling crisis of a disorder." 304.

In our author's experience, blue pill, in doses of four or five grains every night, has appeared to increase the symptoms of dyspepsia, and afterwards when the medicine is discontinued, the symptoms become improved.

"In lesser doses, as of one or two grains every night, continued for a longer time, it has produced no perceptible increase of the symptoms, and is often followed by a more healthy performance of the abdominal functions; which improvement, as the change induced in the action of these organs is gradual, and sometimes complete, is occasionally permanent; or at least continues for some months. By this medicine, in very small doses, the symptoms may sometimes be made to cease under its use; and the disposition which produces them may also be subverted, if the remedy is continued for a sufficient length of time. The former effect, even that of diminishing symptoms, is, however, by no means regular, from blue pill, in any doses: and the latter, that of indisposing organs to a recurrence of the symptoms, is a felicity of treatment which we can seldom boast. Mercury in all its forms is generally prejudicial in the dyspepsia, accompanied by excessive nervous irritability, without evidence of disordered biliary secretion: but even in such cases, an improved state of health, at a more or less distant interval, will generally succeed to its discontinuance, provided it has not been carried to an injurious excess." 307.

He has known a combination of the sulphate of zinc (in doses of a grain twice or thrice a day) with rhubarb and extract of gentian useful in dyspepsia. According to his observations, dyspepsia is sometimes increased, sometimes diminished—or sometimes improved in one respect and deteriorated in another, by a course of purgatives. But when the inconveniences attending purgation have passed away, then an improved state of the digestive organs succeeds. Purgative medicines he has not found successful in mere disorder of the stomach only, with nervous irritability or headaches; "but they are eminently successful if the disorder of the stomach is accompanied with disordered function of the liver, or chronic pain in the side or stomach."

"I have known a fixed pain in the stomach which had existed more than twenty years, and which, for several weeks in each year, suffered one or two exacerbations every day, which terminated in vomiting, perfectly cured, and never afterwards returned, by a course of efficient purgatives, continued for six weeks. As a treatment for affections of this kind, I have found a pill, containing a grain and a half of aloes, one grain of blue pill, and one of ipecacuanha, taken three times a day, a good combination." 309.

Dr. Pring remarks that, in the treatment of any form of chronic disease, whether in the digestive organs, or elsewhere, purgatives frequently increase the symptoms at first—an effect that is rather desirable than otherwise, "inasmuch as it proves that the remedy *has a relation with the disease*," and gives some ground for a hope that a medicine which thus shews itself capable of a præternatural influence on a state of disease, may be capable also of subverting this state, if continued for a sufficient length of time. Dr. Pring has known the symptoms of severe dyspepsia totally cease under the influence of a caustic issue employed for the cure of a local disease. He has also seen benefit derived from the application of adhesive plaster containing six or eight grains of emetic tartar over the region of the stomach. Our Dermalogical writers will not relish the following passage. Without going the whole length which Dr. P. goes, we sincerely believe there is much truth in what he says.—

"The connexion between dyspepsia and diseases of the skin has been before remarked. Those who have written on diseases of the skin, with a great appearance of learning and connoisseurship, have done little more than multiply unnecessary and trivial distinctions, and propose a jargon of barbarous terms, which none but persons of very corrupt taste will take the trouble to remember. I do not, in the works alluded to, remember to have met with any thing like a principle of the pathology or treatment of these diseases. In this place it is necessary only to remark that, as we have seen in our analysis of the relations of disorder of the digestive organs, diseases of the skin are variously connected with such disorder; so a long continued treatment, by small doses of blue pill, perhaps with colocynth, or aloes and ipecacuanha, will cure many of them, without recurring to the more powerful agency of calomel, corrosive sublimate, or arsenic: and, as a local application, hartshorn and water, in the proportion of a dram of the former to an ounce of the latter, is almost a specific in many chronic diseases of the skin, attended either with lymphatic or pustular eruptions, whether confined to one spot, or extending over a whole limb. The effect of this stimulus is to exchange a peculiar for a common inflammation; and, I presume, by the same mode, liquor ammoniæ, liquor potassæ, and turpentine will cure tinea capitis: the proportion of hartshorn is to be regulated by the irritability of the surface, and either applied constantly or occasionally, according to circumstances. This remedy has long been employed in erysipelas, and I have used it with great success in almost every case of disease of the skin described above, which has fallen under this treatment: if continued after the specific character of the disease seems to be subdued, it appears to irritate and produce troublesome exfoliations of the cuticle." 313.

Our author believes that there are very few diseases of the skin that may not be cured—at least he has met with very few. The most effectual of the external applications are sulphur, tar, the different forms of mercurial ointment, hartshorn, zinc, acetate of lead, spirit of turpentine, with oil as a liniment. All these remedies, Dr. P. observes, tend obviously to produce what is called a new action. The internal ones, which chiefly act on the skin, are sulphur, arsenic, ammonia. "These produce heat of skin, temporary fever, and thus substitute an artificial for a natural diseased action." The remedies which cure cutaneous affections, "by their action on related seats," or, in plain terms, by sympathy, are purgatives, emetic tartar, calomel, oxymuriate of mercury, &c.

Of all the modes of obviating habitual costiveness, our author avers, that the most effectual is a residence on the Continent, especially France, where the mode of preparing food is different from that pursued in this country, and where the wines in ordinary use have a tendency to keep the bowels regular. The next best method (as a residence in France is not quite convenient for every one) is a dinner pill composed of from two to six grains of extr. col. comp. It is better mixed with the food than in any other way.

Our author is no advocate for what is called soliciting Nature by endeavouring to have an evacuation every day at a particular hour. We do not think that any force should be used. But by making a gentle attempt at the accustomed hour we shall often succeed—whereas, by going past the hour, the nisus, as it were, goes off, and in torpid states of the bowels, there will be no return of it till the next day.

> "The use of the rectum bougie sometimes disposes to regularity of alvine evacuations. This it does, merely by the stimulus of a foreign body in the rectum, acting as a suppository; an effect which would frequently be accomplished by the presence of feces, if they were allowed to accumulate." 317.

Dr. Pring seems to ridicule the precept of adhering to simplicity of food. "For my own part," says he, "this seems a very trivial matter; for it may be presumed that if fish occupies the inferior, beef the middle part of the stomach, and poultry or game its superior portion, these several articles would be as perfectly digested by the surfaces of the stomach to which they are opposed, as if either sort of food occupied the whole of this viscus." This, we think, is loose reasoning. Dr. Pring seems to forget that by making a dinner of three different kinds of food considerably more *in quantity* will be consumed than if the meal were confined to a single dish—on the well known principle that *variety* stimulates the appetite beyond its natural range. This we consider to be the main evil. But we have great reason to believe that when articles of different degrees of digestibility become mixed in the stomach, they may occasion some inconvenience to that organ, while acting on this compound mass.

And here we must bring our analysis to a conclusion, as the succeeding chapters in Dr. Pring's work are either so abstruse in themselves, or rendered so by the nature of the subjects (principally causation and the occult relation of diseases) that we have despaired of conveying any satisfactory or intelligible information to our readers. We have, therefore, dwelt longer on the plainer and more practical portions of the work, referring those who are curious to a full perusal of the argumentative and doctrinal chapters. As far as we have gone, we think we have done justice to Dr. Pring, and we have no hesitation in bearing our feeble testimony to the genius and talents of our author. That he is not always equal, or even reasonable, is not very wonderful, considering the difficulty of the subject; but we venture to assert that the present work is by far the best which Dr. Pring has published, notwithstanding the extravagant praises that were lavished on a former production.

